# Anthelmintic drug discovery: target identification, screening methods and the role of open science

**DOI:** 10.3762/bjoc.16.105

**Published:** 2020-06-02

**Authors:** Frederick A Partridge, Ruth Forman, Carole J R Bataille, Graham M Wynne, Marina Nick, Angela J Russell, Kathryn J Else, David B Sattelle

**Affiliations:** 1 Centre for Respiratory Biology, UCL Respiratory, Division of Medicine, University College London, Gower Street, London, WC1E 6BT, United Kingdom; 2 The Lydia Becker Institute for Immunology and Inflammation, Faculty of Biology, Medicine and Health, University of Manchester, Oxford Road, Manchester, M13 9PL, United Kingdom; 3 Department of Chemistry, Chemistry Research Laboratory, University of Oxford, 12 Mansfield Road, Oxford, OX1 3TA United Kingdom; 4 Department of Pharmacology, University of Oxford, Mansfield Road, Oxford, OX1 3QT, United Kingdom

**Keywords:** anthelmintic, antiparasitic, cestode, nematode, trematode

## Abstract

Helminths, including cestodes, nematodes and trematodes, are a huge global health burden, infecting hundreds of millions of people. In many cases, existing drugs such as benzimidazoles, diethylcarbamazine, ivermectin and praziquantel are insufficiently efficacious, contraindicated in some populations, or at risk of the development of resistance, thereby impeding progress towards World Health Organization goals to control or eliminate these neglected tropical diseases. However, there has been limited recent progress in developing new drugs for these diseases due to lack of commercial attractiveness, leading to the introduction of novel, more efficient models for drug innovation that attempt to reduce the cost of research and development. Open science aims to achieve this by encouraging collaboration and the sharing of data and resources between organisations. In this review we discuss how open science has been applied to anthelmintic drug discovery. Open resources, including genomic information from many parasites, are enabling the identification of targets for new antiparasitic agents. Phenotypic screening remains important, and there has been much progress in open-source systems for compound screening with parasites, including motility assays but also high content assays with more detailed investigation of helminth physiology. Distributed open science compound screening programs, such as the Medicines for Malaria Venture Pathogen Box, have been successful at facilitating screening in diverse assays against many different parasite pathogens and models. Of the compounds identified so far in these screens, tolfenpyrad, a repurposed insecticide, shows significant promise and there has been much progress in creating more potent and selective derivatives. This work exemplifies how open science approaches can catalyse drug discovery against neglected diseases.

## Review

### The need for anthelmintic drug discovery

Anthelmintic drugs is the collective term for the group of drugs which treat infections of animals or humans infected with parasitic worms (helminths). Parasitic worms infect a wide range of species and as such present a major burden on not only human health, but also livestock production and crop production. There are two major phyla of helminths – nematodes (also known as roundworms) and platyhelminths (also known as flatworms). The nematodes include the soil-transmitted human helminths, e.g., *Ascaris lumbricoides*, and the vector transmitted tissue dwelling filarial worms, e.g., *Wuchereria bancrofti* while the platyhelminths include trematodes (also known as flukes) e.g. *Schistosoma mansoni* and cestodes (also known as tapeworms), e.g., *Taenia solium*.

Although the current review focusses on the unmet need in anthelmintic drug discovery to tackle the burden of human helminth infections, infection of livestock with parasitic worms has important animal welfare implications and can result in considerable economic losses to the livestock industry. In industrialised countries most livestock are routinely given anthelmintics to control or prevent infections and it is estimated the number of sheep, goats and cattle treated annually is hundreds of millions [1]. Treatment of horses, other equids, and companion animals is also a major use of anthelmintics.

Anthelmintic drug discovery has been a continued emphasis in the animal health industry, driven by the spread of resistance to the macrocyclic lactones [2]. In the past 25 years, three new classes of anthelmintic drugs have reached the market: derquantel, emodepside and monepantel. However, the continuing emergence of anthelmintic resistance combined with less predictable infection patterns due to changes in climate have resulted in a breakdown of control of these parasites [3].

Human helminth infections are neglected tropical diseases (NTDs). The most common infections, which we will highlight in this review, are caused by soil transmitted helminths (STHs), schistosomes and lymphatic filarial worms (Table 1). These infections are largely confined to rural, impoverished areas in tropical and subtropical regions of the developing world and co-infections with several different helminths are common [4]. In general, helminth infections are associated with morbidity (see Table 1) rather than mortality and high-intensity infections are associated with increased morbidity. Combined, they represent a massive global burden estimated at 6.4 million disability-adjusted life years (DALYs) [5] with life-long implications as they limit the educational prospects of children and reduce worker productivity [6–7]. Thus, they effectively trap whole countries in poverty.

**Table 1:** Prevalence of and morbidity caused by major human helminth infections. DALYs are disability-adjusted life years.

disease	main etiologic helminth	number infected (million)	DALYs (million)	morbidity

soil-transmitted helminths				

ascariasis	*Ascaris* *lumbricoides*	819 [8–9]	1.3 [5]	infections (due in part to size and number of worms) and intestinal blockages (potentially requiring surgery)_,_ growth stunting and effects on cognition [10–12].
hookworm	*Necator americanus; Ancylostoma duodenale*	439 [8–9]	1.7 [5]	anaemia which can cause complications during pregnancy and post-birth; growth stunting and effects on cognition [13–14].
trichuriasis	*Trichuris trichiura*	465 [8–9]	0.3 [5]	inflammatory foci and haemorrhaging, growth stunting and effects on cognition [15–17].

filarial nematodes				

lymphatic filariasis	*Wuchereria bancroft; Brugia malayi*	120 [18]	1.2 [5]	lymphedema (elephantiasis), hydrocele, renal pathology manifesting as chyluria, and acute dermatolymphangioadenitis causing regular fevers.
onchocerciasis	*Onchocerca volvulus*	20 [19]	1.0 [5]	itching, skin inflammation and visual impairment or blindness

platyhelminth trematodes				

schistosomiasis	*Schistosoma haematobium, Schistosoma mansoni, Schistosoma japonicum*	Over 250 [20]	1.9 [5]	acute infection: myalgia, abdominal pain in the right upper quadrant, diarrhoea, fatigue, malaise, fever, chronic infection: reactions against eggs trapped in host tissues lead to inflammatory and obstructive symptoms; the tissues and organs affected depend on the *Schistosoma* spp. schistosomiasis is also associated with undernutrition, exercise intolerance, diarrhoea (sometimes bloody), chronic pain and anaemia [20].

### Current anthelmintics – STHs

Helminth infections are predominantly located in unseen, rural areas of low income countries; thus despite their prevalence they have been coined the “forgotten diseases of forgotten people” [21]. It is perhaps not a surprise, therefore, that almost all the drugs available for human treatment were initially developed as veterinary medicines.

Effective and safe anthelmintic drugs can reduce prevalence, intensity and morbidity associated with STHs. Two main strategies for delivery exist: the management of diagnosed patients and preventative chemotherapy, which relies on mass scale administration of a single dose treatment to undiagnosed individuals; otherwise known as mass drug administration (MDA) [22–24]. MDA programs are the cornerstone control strategy for these infections and usually target treatment to pre-school or school-attending children.

**Figure 1:**
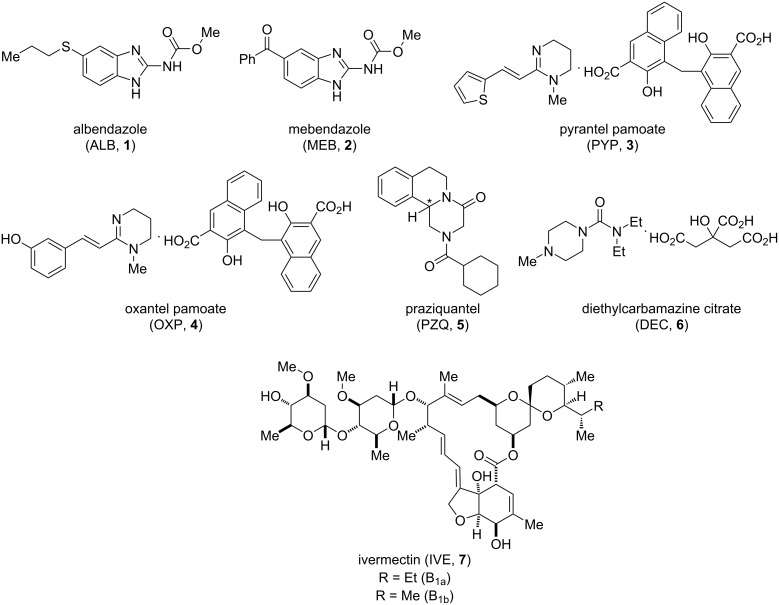
Structures of some current front-line anthelmintics discussed in this review. *Denotes the stereogenic centre: praziquantel is administered as a racemic mixture.

Currently, the World Health Organization (WHO) recommends annual or biannual (where baseline prevalence is over 50%) intervention with the anthelmintics albendazole (ALB, **1**) or mebendazole (MEB, **2**), to treat STHs [25–26]. While achieving cure rates approaching 100% for Ascaris, these drugs, when used as single dose monotherapies, are less effective against hookworm [27–28] and have shockingly poor cure and egg reduction rates against *T. trichiura* [29]. (It is important to note that these drugs do have much better efficacy when administered as a course of treatment. However, given the practicalities and huge scale of mass drug administration programs, single dose efficacy is the benchmark for MDA.) As a result, single dose combination therapies, for example, with the tetrahydropyrimidines pyrantel pamoate (PYP, **3**) and oxantel pamoate (OXP, **4**) have been advocated over recent years with some success (Table 2, Figure 1) [30].

**Table 2:** Drug combinations show increased single-dose cure rates for soil-transmitted helminths. CR: cure rate – the proportion of infected individuals who become negative when tested after treatment with the indicated drug(s). ERR: egg reduction rate – the reduction in egg count after treatment expressed as a proportion of the egg count before treatment. Data from [30].

	MEB (**1**)		ALB (**2**)		ALB (**2**) + PYP (**3**) + OXP (**4**)		MEB (**1**) + PYP (**3**) + OXP (**4**)	
	CR	ERR	CR	ERR	CR	ERR	CR	ERR

*Ascaris lumbricoides*	96.8	99.5	96.5	99.7	90.4	98.3	100.0	100.0
*Necator americanus* and *Ancylostoma duodenale*	41.6	65.1	78.5	92.1	92.8	96.7	84.7	93.4
*Trichuris trichiura*	44.4	80.7	32.1	64.3	84.2	92.7	75.2	90.7

Unfortunately, drug resistance against benzimidazoles **1** and **2** and other anthelmintics have been detected in veterinary parasites, with mutations in the beta-tubulin gene [31–32]. To date there is limited evidence of resistance to benzimidazoles in human STHs [33], although benzimidazole resistance alleles have been found with increased frequency following anthelmintic treatment [34–35]. However, as the use of these anthelmintics has increased in the past decade, thanks to the donation of millions of doses of these drugs to enable mass drug administration, the selective drug pressure on the STHs has increased. This could trigger the emergence of drug resistance [36]. Additionally, the terrestrial stage of STHs can survive as eggs in the soil for several months (*A. lumbricoides* and *T. trichiura*) and larvae several weeks (hookworms), dependent on prevailing environmental conditions [37]. This environmental pool of the infectious lifecycle stage makes even those individuals successfully treated by MDA schemes at risk of reinfection [38]. It has therefore been proposed that, as well as targeting improvements in sanitation to tackle this issue, an environmentally-acting, egg-targeting agent could play a complementary role to help break transmission [39]. The WHO has set a global target to eliminate morbidity due to soil-transmitted helminth disease in children by 2020 through the regular treatment of at least 75% of the children in endemic areas (an estimated total number of 873 million) [40]. It looks unlikely that this target will be met, thus the development of new potent anthelmintic drugs that act via novel mechanisms of action are urgently needed.

### Current anthelmintics – schistosomiasis

Praziquantel (PZQ, **5**), an *N*-acylated tetrahydroisoquinoline-piperazinone derivative has been the basis of schistosomiasis treatment for over 30 years [20]. PZQ is currently administered as a racemic mixture (Figure 1), despite the (*R*)-enantiomer being the biologically active form [41]. As will be discussed later in this review, there has been success in producing the active species in enantiomerically pure form. A clinical trial examining the bioavailability of orally dispersible tablets of levo-praziquantel has been completed [42], and a Phase III trial evaluating safety and efficacy is underway (NCT03845140).

Oral PZQ is safe and efficacious against adult worms of all *Schistosoma* spp., although only very recently have indications of its mechanism of action been advanced [43]. PZQ is the foundation of the global community-based schistosomiasis control programmes which use MDA, to reduce morbidity. A meta-analysis showed that the WHO-recommended dose of PZQ (40 mg/kg) achieved cure rates (CR) of between 76–95% for different *Schistosoma* species and 63.5% for mixed *S. haematobium* and *S. mansoni* infections and mean egg reduction rates (ERR) between 86–95% [44].

However, although PZQ is safe and efficacious against adult worms, it is only given once, and as the drug does not act against the migrating schistosomula stage of the parasite, if these larvae are present a new infection will arise. Moreover, PZQ does not prevent re-infection and therefore transmission can rapidly re-establish from just a few infected patients who can contaminate the aquatic environment.

Additionally, the emergence of drug resistance is a concern with some reports of infections that respond poorly to PZQ in areas where there has historically been heavy use of the drug [45]. However, as is the case for benzimidazoles, there is no clear confirmation that clinically relevant praziquantel resistance has developed [46]. Nevertheless, there is a widespread concern of the risk associated with relying on a single drug, particularly as MDA is being further scaled up, with hundreds of millions of doses of praziquantel being donated every year [20]. Another unknown influence in the future of schistosomiasis treatment is the effect which climate change may have on aquatic environments (and the intermediate freshwater snail host *Biomphalaria glabrata)* and therefore on the distribution of water-borne diseases like schistosomiasis [47].

Similarly to the STHs, the WHO 2020 target for schistosomiasis is morbidity control by reducing the prevalence of heavy-intensity infections to less than 5% amongst school age children. However, whilst these guidelines look likely to be met in lower prevalence regions based on the current WHO MDA guidelines there is a low likelihood that these goals will be achievable in high-prevalence regions where the transmission potential is greater [48].

### Current anthelmintics – lymphatic filariasis

Lymphatic filariasis (LF) treatment has been overseen through the WHO Global Program to Eliminate Lymphatic Filariasis (GPELF) which was launched in 2000. To achieve the WHO goal of the elimination of LF as a public health problem, the GPELF has a two-pronged approach involving not only preventative chemotherapy through MDA to treat the at-risk population, thereby interrupting transmission, but also management of disease morbidity.

By 2018, the GPELF had delivered over seven billion treatments to more than 910 million people [49]. Fourteen countries have eliminated LF as a public health problem, and a further ten countries have been able to stop MDA due to progress in controlling the infection.

MDA for LF involves combinations of three anthelmintics – albendazole (ALB, **1**), diethylcarbamazine citrate (DEC, **6**), ivermectin (IVE, **7**, administered as a mixture of B_1a_ and B_1b_). A recent trial found that a single dose of the triple therapy (IVE + DEC + ALB) was able to clear *W. bancrofti* microfilariae from the blood for three years in almost all treated individuals, and this was superior to a single dose of two drug therapy (DEC + ALB) and non-inferior to three annual doses of the two drug therapy [50]. This trial also provided evidence that, in addition to clearance of microfilariae, both double and triple drug therapies have partial macrofilaricidal effects, as measured by reduction in circulating filarial antigen levels. A second trial also found that a single dose of the three drug (IVE + DEC + ALB) therapy had a greater ability to reduce *W. bancrofti* microfilariae for 24 months after treatment, compared to annual dosing of two drug (IVE + ALB) therapy [51], although microfilarial clearance was not sustained in the treatment population, likely due to reinfection. Importantly, this study also found greater inactivation of adult worm nests (clusters of active adult worms in the lymphatic tissue) in the IVE + DEC + ALB group, demonstrating the macrofilaricidal effect of this treatment combination.

These studies and others have led to WHO recommending triple therapy (IVE + DEC + ALB) for MDA in countries without endemic onchocerciasis or loiasis [52]. Implementation of triple therapy MDA has significant promise for elimination of LF in many countries.

Unfortunately, in countries where onchocerciasis is endemic, DEC (**5**) is contraindicated and achieving a cure is particularly problematic in areas where additionally *Loa loa* is co-endemic and the use of IVE (**7**) is therefore contraindicated. In these circumstances, which apply to some African countries, annual dual therapy (IVE + ALB) or biannual ALB monotherapy are used for MDA as appropriate. Thus, alternative anthelmintics are needed, ideally compounds which can achieve macrofilaricidal (i.e. curative) efficacy but which are safe in regions with onchocerciasis and loiasis.

To this end there has been significant investment into anti-*Wolbachia* treatments. These treatments target the bacterial symbiont *Wolbachia* which is essential for development, growth and survival of many filarial parasites. Targeting of *Wolbachia* with antibiotics has been shown to have curative efficacy against lymphatic filariasis [53] and importantly is safe to administer in *L. loa* co-endemic regions [54]. However, currently available effective antibiotics are unsuitable for public health MDA strategies due to contraindications and treatment duration and therefore novel compounds are required.

### Current anthelmintics – onchocerciasis

Onchocerciasis (river blindness) is caused by infection by *Onchocerca volvulus.* There have been great efforts, beginning in the 1970s, to reduce the burden of this disease, first by control of the insect vector, and later by MDA of donated ivermectin. In 2018, over 150 million people in affected areas received ivermectin [55]. This macrocyclic lactone is an effective microfilaricide but does not kill the adult nematodes. Ivermectin must therefore be administered annually or twice-annually for many years to eliminate the parasite in the population. Progress has been impressive: onchocerciasis has been largely controlled as a public health problem in most of Africa [56], and four countries in the Americas – Columbia, Ecuador, Guatemala and Mexico – have achieved elimination of the parasite [57–58].

There has also been success in attempts to improve MDA for onchocerciasis by repurposing drugs from veterinary medicine. It has recently been shown in a Phase III trial that moxidectin is superior to ivermectin at reducing microfilarial density 12 months after a single dose [59]. This greater duration of action would be expected to reduce parasite transmission between annual rounds of MDA, accelerating progress towards elimination. Emodepside is another veterinary drug that is very promising for repurposing. It has shown activity in pre-clinical models of a variety of human helminth pathogens and is being pursued for treatment of onchocerciasis under an agreement between Bayer and the Drugs for Neglected Diseases Initiative [60]. Phase I safety trials have been completed (NCT03383614).

The management and control of the STHs, schistosomes and filarial parasites relies primarily on chemotherapy and education. Whilst vaccines are being developed for roundworms and whipworms, the development is still at the pre-clinical stage [61–65]. A hookworm vaccine is at a more advanced stage of development [66] and a schistosomiasis vaccine is in a clinical Phase III trial [67], however, no vaccines are currently in use in the field.

The current anthelmintic pipeline and the drug discovery landscape for parasitic helminth infections is sparse. This contrasts with the situation for malaria and kinetoplastid infections, where efforts, particularly by the Drug for Neglected Diseases Initiative and Medicines for Malaria Venture, in partnership with various pharmaceutical companies, are now paying off with an improved pipeline of drug development and the approval of tafenoquine (**11**) and fexinadole (**10**) [68–69]. With increasing concerns over the potential emergence of resistance to currently deployed anthelmintics, the possibility of climate change altering the distribution of these parasites, coupled with the inability of the currently available chemotherapies to impact parasite transmission and the poor efficacies of some of these drugs, e.g. against *Trichuriasis*, the need for new approaches to anthelmintic development is pressing. Additionally, the majority of anthelmintics are limited by their poor cross-phyla activity, e.g., praziquantel (PZQ, **5**) has efficacy against trematodes and cestodes but not nematodes. Only the benzimidazoles **1** and **2** show some broader effects but are much more active against nematodes than against cestodes or trematodes [70]. Ideally an anthelmintic with broad activity against different helminth infections would be desirable, although this may be too much to hope for given the evolutionary distance between the different target phyla.

As it stands, the WHO roadmap on NTDs, which set out a comprehensive plan for the control, elimination and eradication of NTDs, looks unlikely to deliver the desired outcomes by 2020. As the NTD 2030 roadmap is being rolled out there is an urgent need for novel anthelmintics to enable eradication of these diseases of poverty.

### Application of open science to anthelmintic development

#### Commercial incentives for anthelmintic development

Despite the important need for new drugs and other control solutions for human helminth infection, these indications have been largely ignored by the pharmaceutical industry, presumably for commercial reasons. Indeed, no new chemical entities were approved between 2000 and 2011 [71–72]. Since then only moxidectin (**8**, 2018) and triclabendazole (**9**, 2019) have been approved. The major drugs used for control of human helminth infections have been in clinical use for many years: ivermectin (**7**, FDA approval in 1996), mebendazole (**2**, 1974), albendazole (**1**, 1996), praziquantel (**5**, 1982), diethylcarbamazine (**6**, 1950). The scarcity of new drugs reflects the limited economic incentive to spur commercial investment in neglected tropical diseases such as human helminth infection.

**Figure 2:**
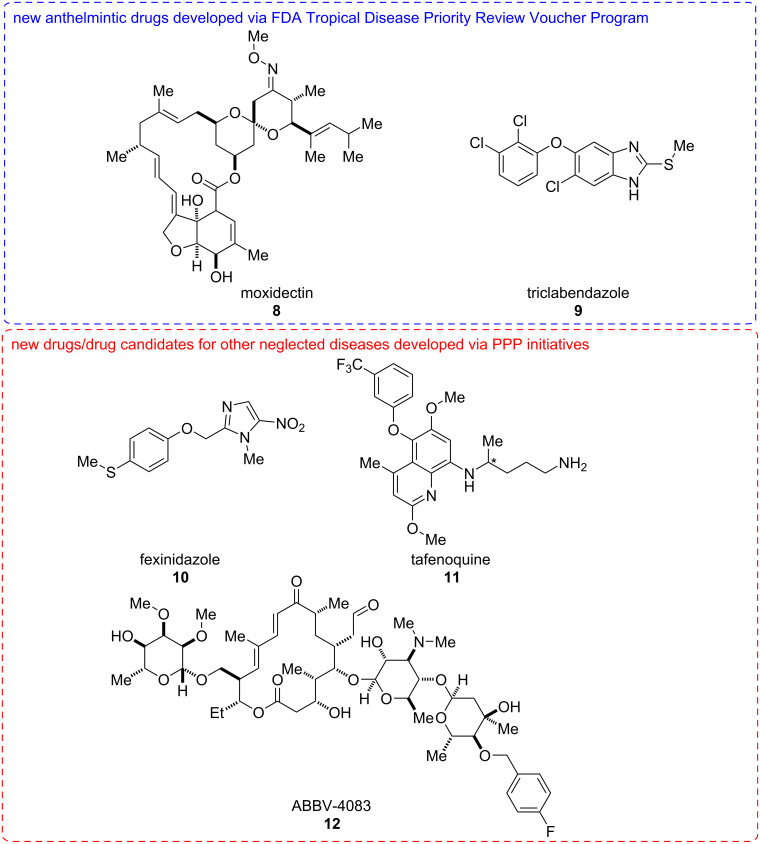
Structures of new anthelmintics drugs developed through repurposing, and new drugs or drug candidates for other neglected diseases developed through public–private-partnership (PPP) initiatives. *Denotes the stereogenic centre: tafenoquine (**11**) is administered as a racemic mixture.

Efforts have been made to promote such investments. The FDA Tropical Disease Priority Review Voucher Program aims to create a commercial incentive to develop new drugs for otherwise neglected diseases [73–74]. Organisations that have an eligible drug successfully approved receive a transferrable voucher for a further priority review that has substantial value. In recent years two anthelmintic drugs have received the support of this program: moxidectin (**8**) showing superiority to ivermectin (**7**) for onchocerciasis [59], and triclabendazole (**9**) approved for fascioliasis, although both drugs were originally developed for veterinary indications and triclabendazole (**9**) was used for the treatment of fascioliasis for many years before FDA approval associated with the voucher program (Figure 2).

Public-private partnerships (PPPs), which typically bring together diverse organisations such as pharmaceutical companies, governments and charitable organisations are now proving successful at bringing drugs through to approval for neglected tropical diseases. For example, fexinidazole (**10**) was developed by the non-profit Drugs for Neglected Disease Initiative, in partnership with Sanofi, the Swiss Tropical and Public Health Institute, and other organisations. It has now been approved as the first all-oral treatment for all stages of human African trypanosomiasis [69,75]. A partnership between Medicines for Malaria Venture and GSK developed tafenoquine (**11**), which is effective as a single-dose treatment for the radical cure of *Plasmodium vivax* malaria [68]. While neither of these examples is for helminth infection, they show that these organisational models can be successful at bringing new molecules through to clinical practice. At an earlier stage of development, the anti-*Wolbachia* (A·WOL) consortium, a partnership including the Liverpool School of Tropical Medicine and AbbVie discovered ABBV-4083 (**12**), an antibiotic effective in preclinical models as a macrofilaricide by acting on the *Wolbachia* bacterial endosymbiont [76] (Figure 2). WIPO Re:Search is another public–private partnership that facilitates work in neglected tropical diseases by bringing together intellectual property, expertise, facilities and funding from pharmaceutical companies, universities, and non-profit organisations. The number and breadth of projects, including many in the area of helminthiasis, that have been facilitated by this organisation since it was founded in 2011 is impressive [77].

#### Open science: an efficient model for drug innovation

An alternative way to promote anthelmintic drug discovery is to reduce the cost, by introducing research strategies that make drug innovation more efficient [72]. Open source drug discovery is a model that seeks to completely open up the research process [78]. This has several radical advantages that challenge traditional drug discovery. Secrecy and the hoarding of data in silos, such as individual research groups waiting for publication of their data, stifle our ability to access the best ideas. Openness can create communities that collaborate and attract new expertise when needed or serendipitously create new directions as different people from around the world and different fields bring fresh insights. Timely sharing of data speeds up research and avoids inadvertent repetition of effort. The wider drug discovery community is gradually adapting to these types of challenges, with notable examples being ChEMBL (European Molecular Biology Laboratory) and PubChem (NIH). Both focus on characterising drug-like molecules and providing information to the public domain. The former is a manually curated database of bioactive molecules, which aims to bring together chemical, bioactivity and genomic data to aid the translation of genomic information into effective new drugs [79]. The latter is an open database for researchers to upload scientific data, including biological results, so that others may use it [80].

Perhaps the biggest example of open source drug discovery in its pure form is the Open Source Malaria project [81–82]. This is a platform for malaria-related research, with an emphasis on drug development. At the heart of this project are the completely open online electronic lab notebooks, that immediately share all work being done on the various strands of research of the project. Results are shared and publicly discussed, and priorities set on the Github issues page of the project. Importantly, anyone is free to jump in with suggestions, and indeed the Open Source Malaria Project has been successful at receiving high-quality contributions, from a wide range of sources. A highlight of this work has been the detailed exploration of an arylpyrrole antimalarial series [82]. We are not aware of a similar real-time, fully open source effort being applied to anthelmintic discovery, but this would be an exciting prospect for the field. However, researchers have been freely releasing open tools useful for drug discovery, openly describing their compound screening efforts, and participating in distributed open library screening projects such as the Medicines for Malaria Venture Pathogen Box. The point has recently been made by Tim Geary and colleagues that millions of compounds have been screened in industrial and academic labs on isolated helminths, but that the rate of drug discovery has been very low, so efforts must be made to enhance cooperation among the various groups pursuing this strategy [83]. They go on to suggest that sharing of both positive and negative screening data via online databases is a priority to focus attention on the most promising compounds and to reduce the redundancy of effort. Such a collaborative data-sharing structure must be a priority for the field.

### *C. elegans:* a model organism for parasitology and an exemplar of an open community

#### *C. elegans* as a model nematode

*Caenorhabditis elegans* (*C. elegans*) is a non-parasitic nematode worm that is found worldwide and was selected by Sydney Brenner as a genetic model organism for biological research with strong potential to contribute to our understanding of developmental biology and neurobiology [84]. In 1998 it became the first complex eukaryote to have its genome sequenced [85]. *C. elegans* occurs as hermaphrodites and males and its capacity for hermaphroditic reproduction (selfing) facilitates the long-term maintenance of genetic strains. The capacity to freeze and store strains in glycerol adds to its utility. The transparency of the worm facilitates studies on the development, and *C. elegans* remains the only complex organism for which the entire cell lineage has been described [86]. For this pioneering work, Brenner, Horvitz and Sulston were awarded the 2002 Nobel Prize in Physiology/Medicine. The nervous system, which makes up 358 of the hermaphrodite’s 959 somatic cells, is the only one for which a complete wiring diagram is known [87], facilitating studies on neural signalling and nervous and neuromuscular disorders [88] as well as research in understanding the anthelmintic drug action [89–90].

To grow and maintain *C. elegans* in the laboratory is relatively straightforward. Their small size (1 mm in length as adults) means ease of storage. Their rapid life cycle (approximately 3 days from egg to adult), and short lifespan (approximately 2–3 weeks) when fed on a diet of *E. coli* facilitates genetic studies. Forward and reverse genetics are pursued conveniently in *C. elegans*. A rich diversity of mutants is available via the Caenorhabditis Genetics Centre [91]. The discovery of RNA interference delivered via feeding worms double-stranded DNA [92] has opened the door to genome-scale gene knockdown in the search for new drug targets. These approaches can expedite the validation of drug targets and the identification of new candidate molecular targets.

So how can a free-living worm contribute to our understanding of parasitic nematodes and the development of anthelmintic drugs? A key advantage is the ease of culture of *C. elegans*. Large numbers can be generated rapidly and at low cost which enables high-throughput chemical and genetic screening studies. It is often difficult or impossible to undertake comparable studies on parasitic worms due to the challenges of maintaining parasitic worms outside their host, although rodent models are available for many classes of helminth [93].

Although *C. elegans* is clearly not a target organism, it can be deployed in the search for new anthelmintics for animal health and human health applications. Screens can be pursued for new chemical leads which may then be applied to other parasitic species. *C. elegans* chemistry-to-gene screens, facilitating deconvolution of the molecular target and mechanism of action, are also useful. For example, Burns and colleagues screened 67,012 compounds to identify those that kill *C. elegans* and followed this by rescreening hits in two parasitic nematode species and two vertebrate models (HEK293 cells and zebrafish). By this means, they identified 30 structurally distinct anthelmintic lead molecules [94]. They also determined the target (complex II of the electron transport chain) of one lead compound, that showed nematode specificity and nanomolar potency. This work shows that *C. elegans* can be effective, cost-efficient, and has a role to play in the anthelmintic drug discovery process.

Another potential attribute in the context of investigating parasites is the ease with which the *C. elegans* genome can be manipulated, enabling the generation of transgenic *C. elegans* expressing anthelmintic drug targets from a parasitic worm [95–96]. These approaches are still in their infancy, but such genetic modifications can give rise to scorable phenotypes reflecting the properties of the parasite drug target which may in future lend themselves to high-throughput chemical and genetic (RNAi) screening approaches.

There are, however, limitations to using *C. elegans* as a research tool, notably its innate physical and enzymatic defences to xenobiotics, factors important for the survival in its natural environment. As a result, *C. elegans* is somewhat inaccessible to some chemicals, meaning that high concentrations of certain compounds may be required to observe changes in the phenotype [90,97].

**Figure 3:**
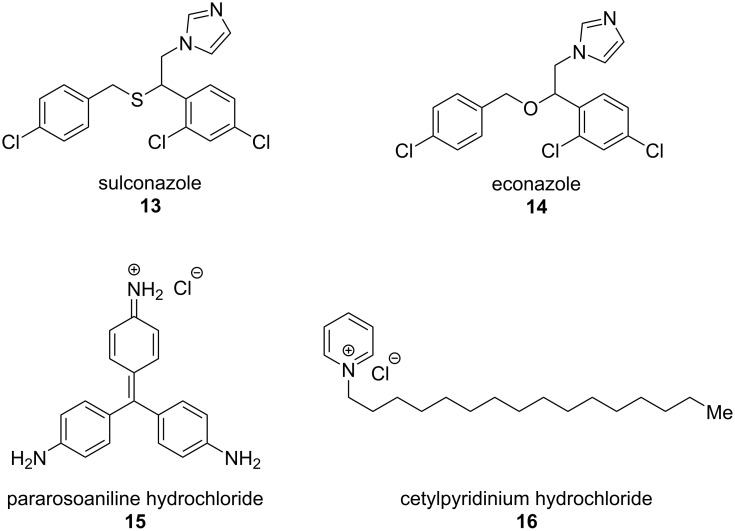
Compounds with anthelmintic activity identified by a combination of screening against *Ancylostoma ceylanicum, C. elegans, and T. muris* [98].

Aroian and colleagues [98], in line with the work of Burns et al. [94], counsel caution on relying on data from *C. elegans* alone, which makes perfect sense as it is never the primary target organism. They screened a compound library against both adult and free-living larval stages (egg to L3i larval development assay, E2L) of the human hookworm parasite *Ancylostoma ceylanicum* and against *C. elegans*. They found that the *A. ceylanicum* E2L assay was more successful at identifying compounds active against *A. ceylanicum* adults than *C. elegans* assays (lower false negative rate). This works lead to the testing of four compounds with in vitro activity in an in vivo *A. ceylanicum* hamster infection model – sulconazole (**13**), econazole (**14**), pararosaniline (**15**) and cetylpyridinium chloride (**16**) (Figure 3). Of these pararosaniline (**15**) showed a significant reduction in parasite egg production in this model, despite no activity in *C. elegans* assays.

Perhaps one of the best reasons for integrating *C. elegans* into the discovery process is that if interesting new molecules are identified which are active on both the target parasite and the genetic model organism, but the precise target remains unclear, then *C. elegans* genetics can offer a route to target identification that would be difficult by any other route.

#### The *C. elegans* community: historically an open science model

Research into *C. elegans* was pioneered by researchers including Victor Nigon, Ellsworth Dougherty and Jean-Louis Brun [99]. However, the use of *C. elegans* as a model organism in fields such as genetics, developmental biology and neuroscience was established by Sydney Brenner in the 1960s at the MRC Laboratory of Molecular Biology in Cambridge, UK [100]. An important feature of *C. elegans* research has been the community of researchers, with a long tradition of openness and the sharing of ideas and reagents, which we today can recognise as an example of open science [101].

This openness is exemplified by a newsletter for *C. elegans* researchers, *The Worm Breeder’s Gazette,* which combines advice and suggestions on methods with informal communication of new findings in advance of publication. For example, the Nobel prize-winning use of a green fluorescence protein as a marker was reported in the *Gazette* by Martin Chalfie and colleagues five months before the peer-reviewed publication [101–103].

A second example of the history of *C. elegans* open science is the genetic map and genome sequencing project, led by John Sulston and Bob Waterston, which made *C. elegans* in 1998 the first multicellular organism to have its genome sequenced. The project involved teams at the Genome Sequencing Center at the Washington University School of Medicine (St Louis, Missouri, USA) and the Sanger Centre (Hinxton, Cambridge, UK) sharing a belief that ‘together, we can do more’, rather than ‘one against the other’ [104]. An important feature was that genomic clones were made freely available, enabling researchers to investigate genes of interest [101]. Furthermore, genetic and genomic information was rapidly distributed, in the form of the ACeDB database, initially via gopher, an early internet service, and later via WormBase [105–106].

These historical examples illustrate the strength of open science in the *C. elegans* research tradition. In the following sections of this review we discuss how open science approaches continue to be important in the field of anthelmintic and antiparasitic drug discovery.

### Open approaches to target identification

Genomic resources are important for target identification, particularly in the case of parasites, as the life stages found in the host are often difficult to obtain or culture, and few molecular tools are available. WormBase ParaSite [107] is an important central resource for helminth genomic data [108]. At the time of writing, this database contains information on 142 species of parasites and other helminths, including genomes, comparative genomics data and RNAseq studies, along with a number of tools facilitating access to this data including a genome browser, BioMart (a tool for exporting tables of selected information) and a REST API, an interface for programmatic access to the database.

But how can we get from genomic information to new drug targets? A recent comparative genomics study from a large international consortium of researchers has really helped with this question, by comparing the genomes of 81 species of parasitic and non-parasitic worms (both nematodes and platyhelminths) [109]. This work produced large open datasets, such as expanded gene families relevant to parasitism, and analyses of the metabolism in different parasites across the phyla, important for exploring metabolism in the search for new drugs. Furthermore, this study predicted promising anthelmintic targets and compounds likely to interact with these targets, thereby identifying drugs for potential repurposing as anthelmintics.

Once targets have been identified, it is desirable to obtain genetic/pharmacological proof-of-concept for target validation. Whilst RNA interference and CRISPR methodologies are now being applied to parasites themselves [110–112], inevitably, large-scale functional genomic resources are mainly found in *C. elegans.*

The *C. elegans* Gene Knockout Consortium has obtained putative knockout mutations in around 15,000 genes, and has now adopted CRISPR/Cas9 to extend the resource to every gene in the genome [113]. A complementary collection of knockout mutants from the National Bioresource Project in Japan is also available [114]. Both projects make the mutants openly available for low cost.

Another open source resource with immediate applicability to target identification and validation is the Open Worm Movement Database [115]. This is an open platform for analysing and sharing worm behavioural data, such as that obtained from worm tracking software. For example, the researcher can search for worm strains with a particular movement phenotype, such as low movement speed. These paralysed or poorly moving worm mutants may be a source of novel neuromuscular anthelmintic targets. The researcher can immediately view videos of the identified worm strains on YouTube to confirm their hypothesis.

### Open tools for phenotypic screening

Recently, many laboratories throughout the world have recognised the need for the development of new anthelmintic compounds, so have initiated screening programmes against various pathogens and models. Despite our growing knowledge about potential targets for new anthelmintics, phenotypic screens involving assays of parasites or models in vitro remain important [83]. As a result, several methods and screening platforms have been developed to improve the screening speed and reliability.

In this section we discuss recent phenotyping methods and systems, and how they have been applied to anthelmintic discovery. We concentrate on those where the software source code and/or hardware design is made clearly and openly available. Methods, applications, and location of source code/design are summarised in Table 3.

#### Open tools for phenotypic screening of motility and viability

Several groups have developed image acquisition and analysis systems for high-throughput phenotypic screening of parasites (Table 3), typically using the approach of thresholding difference images/movies to quantify motility, sometimes segmenting the image by recognising the organism of interest [116].

**Table 3:** Open tools for high-throughput phenotypic screening of motility and viability and their use for anthelmintic discovery.

tool	validated with	source code/description (license)

WormScan [117] automated WormScan [118]	*C. elegans* high-throughput screen [119]	paper supporting information [117–118]
Lifespan Machine [120]	*C. elegans* lifespan analysis	Github [121] (GPLv3)
WormAssay [122] Worminator [123]	*Brugia malayi* (adults and microfilariae)*, Cooperia* spp. L3, *Dirofilaria immitis* microfilariae*, Schistosoma mansoni* [122–123]	Github [124] (GPLv2 or later)
Cestode motility ImageJ macro [125]	*Echinococcus multilocularis* protoscoleces	paper supporting information [125]
Wiggle index ImageJ macro [126–128]	multiple high-throughput library screens with *H. contortus* [129–134]	paper supporting information [126]
INVAPP paragon [135]	library screens with *T. muris* and *C. elegans* [39,135–137]	Github [138] (MIT license)
CellProfiler [139] CellProfiler schistosome pipeline [140] CellProfiler WormToolbox [141]	*S. mansoni* thioredoxin glutathione reductase inhibitor screening [140], *C. elegans* live/dead high-throughput screening [141]	Github [142] (BSD license) CellProfiler published pipelines website [143]

WormScan is a method that uses a flatbed scanner to capture sequential images, where the scanner high-intensity light usefully stimulates the worm movement [117]. This system has been utilised to screen a 26000 compound library in a *C. elegans* growth assay [119]. An updated version of this software (Automated WormScan) has recently been published [118]. The Lifespan Machine also uses a scanner to acquire images, and has the ability to monitor thousands of worms simultaneously and determine mortality time for individual worms on plates [120].

WormAssay is a combination of a video camera and an open source software package. It uses two algorithms (Lucas–Kanade optical flow estimation, and a pixel change method) to determine the motility of macroparasites in microtitre plates [122]. The Worminator builds on WormAssay for the use with microscopic parasites [123]. This system has been validated for determining the anthelmintic activity against a variety of nematodes and schistosomes. A screening using the Worminator identified auranofin as a promising candidate for repurposing as a treatment for of lymphatic filariasis and onchocerciasis [144]. This drug was originally approved as a treatment for rheumatoid arthritis but is currently in trials for amoebiasis and giardiasis (NCT02736968).

An ImageJ [145] macro that determines the motility of *Echinococcus multilocularis* protoscoleces within microtitre plates by pixel difference thresholding has been used to identify an anthelmintic hit compound MMV665807 [125]. Wiggle Index, another ImageJ macro for difference thresholding and motility quantification has been extensively used for library-scale screening of exsheathed *Haemonchus contortus* L3s [127–130]. INVAPP Paragon, an imaging setup and MATLAB analysis script that again uses difference thresholding for motility quantification has been used for library screening with *Trichuris muris* and *C. elegans* [39,135–136].

**Figure 4:**
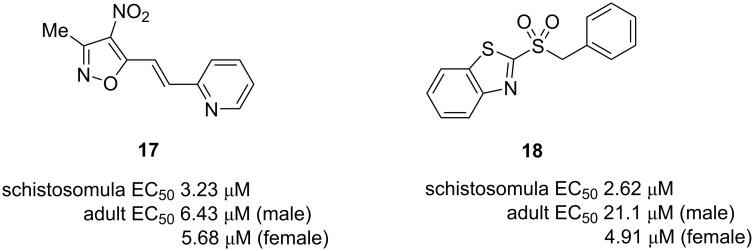
Inhibitors of *S. mansoni* thioredoxin glutathione reductase with anthelmintic activity [140].

CellProfiler is a major open source package for quantitatively measuring phenotypes from imaging data, particularly from high-throughput screens [139]. Some groups have developed helminth analysis methods using CellProfiler. A virtual screening approach was used to identify inhibitors of *S. mansoni* thioredoxin glutathione reductase [140]. These virtual hits were then tested in a CellProfiler-based high content screen using *S. mansoni* schistosomula, which determines both the motility and a range of other phenotype scores, leading to the identification of 2 new small molecules with distinct chemical scaffolds **17** and **18** with activity against schistosomula and adult worms at low micromolar concentrations (Figure 4). Another CellProfiler toolbox enables the quantitation of *C. elegans* viability and fluorescence [141].

#### Open tools for more detailed analysis of helminth physiology

Sophisticated software and hardware methods have been developed to phenotype more subtle aspects of worm biology than paralysis/motility/viability assays. These methods could form a fruitful basis for finding compounds that are anthelmintic in vivo but do not cause paralysis, perhaps involving aspects of the interaction with the host, interfering with the secretion of proteins, or other ways of damaging the worm [146]. They are also useful for understanding in more detail the mechanism of action of anthelmintic compounds, since it has been recognised that we do not fully understand how many anthelmintics work – for example the concentrations of macrocyclic lactones that paralyse worms in vitro are much greater than the concentration achieved by effective doses in vivo [147].

Several different open source systems have been established for tracking the worm movement of *C. elegans* [148–151]. These systems typically measure a number of parameters in addition to speed such as bending, reversals, and other aspects of behaviour. CeleST is a similar open source quantitative locomotion analysis system that measures aspects of nematode swim behaviour [152]. Such systems have, to our knowledge, not been utilised with helminth parasites, but such studies would be fruitful to dissect anthelmintic actions in detail.

Microfluidic systems have great potential to aid anthelmintic discovery by enabling finely detailed individual worm longitudinal microscopy. They have the potential to greatly reduce the amount of compound required for a screen hence enabling larger libraries to be economically screened. Encouragingly, some authors have made their microfluidic chip designs openly available, enabling utilisation and modification by other groups. Stress-Chip is a chip that allows the isolation of a hundred worms in single-worm arenas and monitoring as chemicals flow over the worms [153]. The CAD file for producing the microfluidic device has been made available on Figshare under the CC BY 4.0 license [154]. Another 10-chamber worm isolation microfluidic device has been reported, originally for the imaging of worms to quantify the sleep behaviour during development, and the CAD file is made available in the supporting information [155].

The cost of the equipment is of course often a concern, especially for groups working on neglected tropical diseases and/or in developing countries. Recently, an open hardware project has reported Incu-Stream, a long-term imaging system capable of automatically scanning wells across microplates and recording videos of worm movement for further analysis [156]. The authors provided a parts list with a total materials cost of $184. Schematics, CAD files and the associated software are provided on Github [157].

### Open approaches to developing therapeutics

#### The Pathogen Box project

The Pathogen Box is a 400 compound collection that was made freely available by the Medicines for Malaria Venture (MMV), a not-for-profit product development partnership organisation [158]. The compounds have demonstrated activity against a variety of neglected tropical disease pathogens [159]. This is an open access science project, with the only condition that researchers agree to share their results. This model follows on from the successful MMV Malaria Box, where 55 groups compiled results from over 290 different assays in a diversity of screens related not only to malaria, but also to other neglected tropical diseases [160].

**Figure 5:**
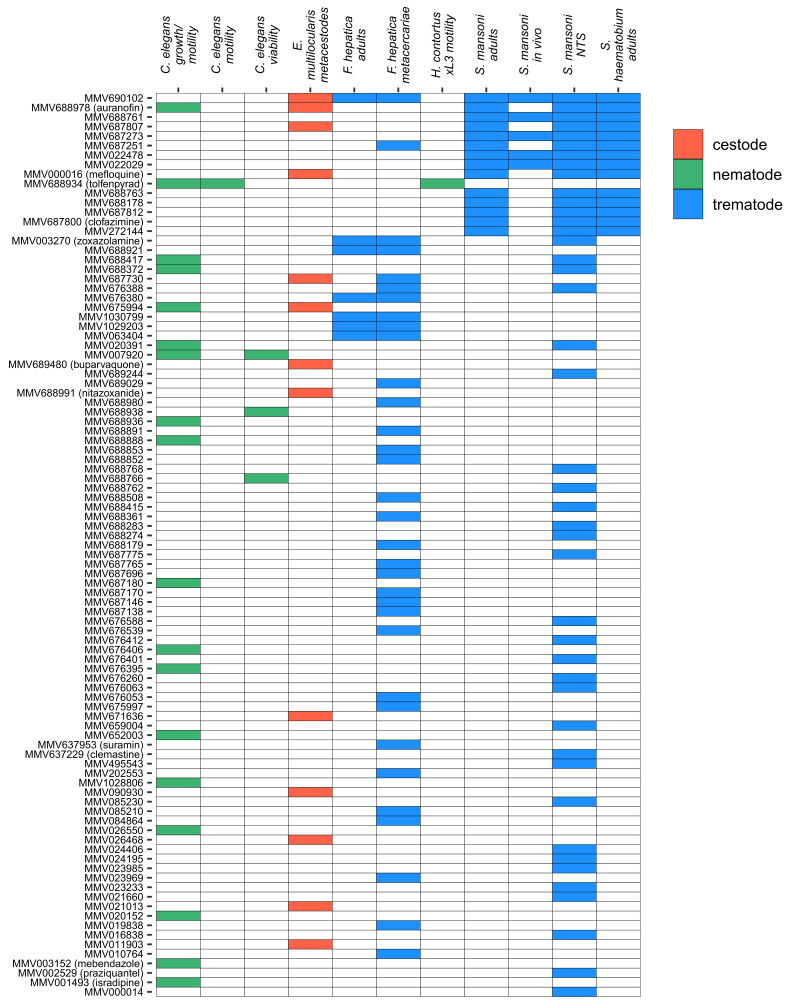
Active compounds from anthelmintic screens using the MMV Pathogen Box. NTS: newly transformed schistosomula, xL3: exsheathed L3. Assay outline and citations for data sources: *C. elegans* growth/motility screen – automated quantification using the INVAPP system [135]. *C. elegans* motility screen – automated quantification using the WMicrotracker ONE system [163]. *C. elegans* viability screen – automated quantification of viability by differential absorption of the dyes DB-1 and propidium iodide [164]. *E. multilocularis* metacestode screen – damage was assessed by quantifying the activity of phosphoglucose isomerase released into the culture media [162]. *F. hepatica* screens – viability of metacercariae was assessed using a scoring system taking into account membrane damage and fluke translucency, and viability of adults was assessed using a scoring system taking into account worm motility, colour and rigidity [165]. *H. contortus* screen – automated quantification of motility of exsheathed L3 worms [129], *S. haematobium* and *S. mansoni* screens – activity was assessed using a scoring system taking into account motility and morphological/tegumental changes [161].

The Pathogen Box project is currently active, but several groups have already reported anthelmintic screens using this library [129,135,161–165]. We have compiled the results from these published screens in Figure 5. These results already highlight how the open approach enables the library to be tested against a variety of different organisms, enabling researchers to identify and prioritise compounds active against multiple pathogens. For example MMV690102, which was originally developed as an inhibitor of kinetoplastid dihydrofolate reductase [159], is active against three trematode species (*F. hepatica, S. haematobium, S. mansoni* [161]) and the cestode *E. multilocularis* [162].

Perhaps the most promising lead from the Pathogen Box so far is tolfenpyrad, a pyrazole-5-carboxamide insecticide, which was first identified as an anthelmintic with activity against exsheathed L3 and L4 parasitic life stages of *Haemonchus contortus,* a major parasite of ruminants [129]. Subsequent studies have demonstrated activity against the model nematode *C. elegans* [135,163]. Tolfenpyrad (**19**) was found to be highly potent, with an IC_50_ value between 0.02 and 3 µM in various *H. contortus* assays and 0.2 µM in a *C. elegans* assay [129,135]. A follow-up study identified two additional pyrazole-5-carboxamide compounds with activity against *H. contortus,* although not improving on the potency of tolfenpyrad [166]. Tolfenpyrad acts in arthropods as an inhibitor of mitochondrial complex I [167]. It will be interesting if a tolfenpyrad derivative can progress to trials as it would be a new mechanism of action for an anthelmintic, although some mitochondrial uncouplers, such as the veterinary medicine closantel, are active against *Fasciola hepatica* [168].

Recently, a medicinal chemistry effort was undertaken to determine the anthelmintic structure–activity relationships for tolfenpyrad (**19**) [169]. The main objective of this work was to reduce the lipophilicity of tolfenpyrad **19**, which was considered undesirable for an orally administered agent, as typical of anthelmintics, compared to a surface-applied pesticide. This was accomplished through systematic alteration of the pyrazole-5-carboxamide and phenoxybenzyloxy moieties within tolfenpyrad **19** (Table 4).

**Table 4:** Potent anthelmintic activity of tolfenpyrad (**19**) derivatives against *H. contortus.* The activity is shown for two in vitro assays: one for motility of xL3 (exsheathed L3 stage worms) and a second for development of xL3 into the L4 stage [169].

ID	structure	IC_50_ (μM) in xL3 motility assay	IC_50_ (μM) in L4 development assay

**19** (tolfenpyrad)	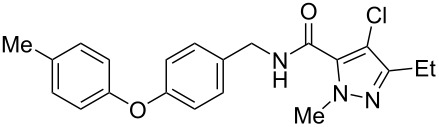	2.9	0.03
**20**	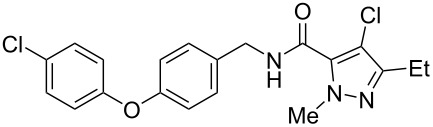	2.03	0.0008
**21**	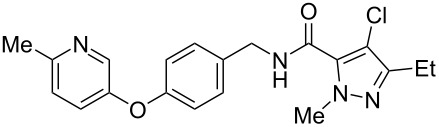	4.0	0.03
**22**	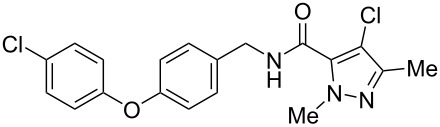	3.33	0.01
**23**	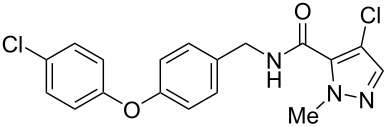	0.38	0.0007
**24**	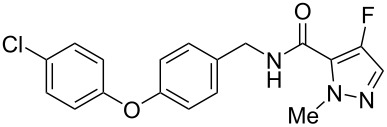	0.7	0.0008

The systematic variation of the *p*-methylphenyl ring within **19** gave rise to a number of aromatic and heteroaromatic analogues with similar levels of potency to tolfenpyrad (Table 4). For instance, replacement of a methyl group with a chlorine atom in **20** maintained similar levels of potency in both the xL3 motility assay and the L4 development assay. Similarly, replacement of *p*-methylphenyl group with a 2-methylpyrid-5-yl group in **21** largely maintained potency whilst lowering lipophilicity. Conversely, modifications to the pyrazole group gave rise to more dramatic changes in the potency. For instance, while changing the ethyl substituent within **20** to a methyl substituent in **22** gave a slight increase in potency, removal of the ethyl group in **23** showed a substantial improvement in the activity, with IC_50_ value for the xL3 motility improved to 0.38 µM and for the L4 development to 0.7 nM, with a similar activity for the corresponding fluoropyrazole derivative **24**. These latter two compounds **23** and **24** showed high selectivity for the parasite, with low or no cytotoxicity. The authors went on to demonstrate that **24** showed a broad activity against other nematode parasite models: *H. polygyrus, A. ceylanicum* and *T. muris.* A broadly-related 1-methyl-1*H*-pyrazole-5-carboxamide series has also been investigated in detail, with compounds identified that show substantially improved potency and selectivity compared to tolfenpyrad [131,170].

#### Praziquantel (**5**)

Schistosomiasis is a major tropical disease resulting from the infection by a trematode parasite, the blood fluke *Schistosoma mansoni* [171]. After malaria, it is the next most devastating parasitic disease with millions affected worldwide. No vaccine is available but the drug praziquantel (**5**) is an effective treatment. It is administered to children or whole communities often in mass drug administration (MDA) programmes [172]. An unfortunate drawback is that the drug is currently generated and administered as a racemic mixture. The pure active enantiomer would be preferable for several reasons, for example, the inactive enantiomer has been linked to unwanted side-effects and also contributes a very bitter taste.

**Figure 6:**
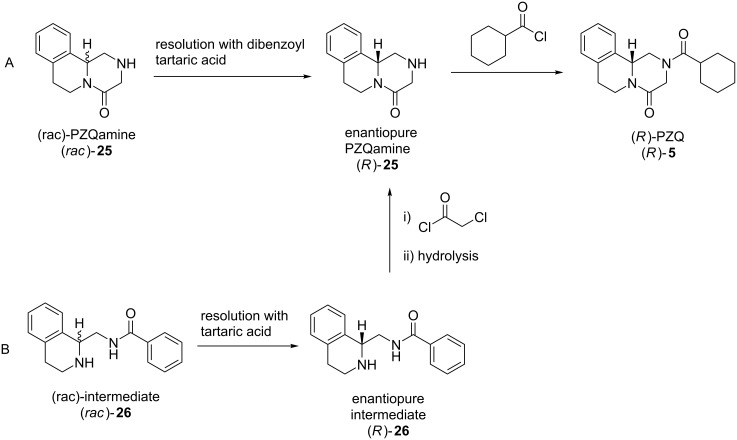
Two resolution approaches to enantiopure PZQ (*R*)-**5** discovered through A) open science and B) contract research [173].

With a view to finding a synthetic route to the active enantiomer, an open website was established, and several groups became involved, both academic and commercial laboratories. As a result, two different approaches to the problem emerged where hitherto there had been none (Figure 6). The hydrolysis to an intermediate amine **25** which was then resolved with a derivative of tartaric acid was a solution that emerged from this open source approach. Another solution was identified by a sponsored contract research team. This involved a different intermediate **26** which was then, in turn, resolved using tartaric acid itself. A detailed account of the successful resolution process has been published by Matthew Todd and colleagues [173]. This has not yet led to the pure enantiomer being widely available, but the setting up of an open science project was the stimulus to the solution of a challenging problem. A Phase III clinical trial testing safety and efficacy of ʟ-praziquantel is currently recruiting (NCT03845140).

## Conclusion

The recent years have seen unprecedented investment and effort to distribute anthelmintic drugs to millions of people in mass drug administration programmes. Onchocerciasis has been eliminated from four countries in the Americas [57–58], with the prospect of elimination from African countries in the coming decade. These advances have been made with our existing major anthelmintic drugs. In addition, there has been great progress in finding more effective combinations of drugs, as well as bringing forward trials of veterinary anthelmintics to combat human disease. However, the risk of mass drug administration leading to resistance, as well as the growing understanding that existing drugs are not ideal for all human helminth infections, has led to new focus on the need to develop new anthelmintics. Unfortunately, there is limited economic incentive to fund drug development adequately.

Open source science seeks to radically open up the drug development process, with the goal of increasing the efficiency, reducing the cost of research duplication, reducing the hoarding of data and creating collaborative communities [174]. This approach has been applied to the discovery of compounds with antimalarial activity [82]. In the field of anthelmintic drug discovery there is much open science. In this review we particularly highlight open-source assay systems that have been developed and openly released by several groups that enabled compound screening against different helminth parasites. The genomic information about helminths is rapidly expanding and most is released freely and can be queried by scientists around the world, helping to find new anthelmintic drug targets. Probably the weakest area for open science is compound screening and subsequent drug development. The authors of this review are probably as guilty as other members of the community. Despite having good intentions and releasing screening data with publications, we could all do more to release data soon after collection, rather than being constrained by academic and publishing timescales. The MMV Pathogen box has demonstrated how many different groups around the world can be recruited to screen compound libraries in their specialist assays, and release data in a relatively timely manner – researchers are asked, as a condition of receiving the compounds, to share any data generated in the public domain within two years.

What are the barriers to making anthelmintic drug discovery more open? Perhaps more could be done to facilitate data sharing. While existing databases such as PubChem and ChEMBL can be used to share screening data, a specialised anthelmintic screening and target database could promote more widespread use and release of data in a consistent and accessible format, enabling more collaborative working and reuse of data. Wider awareness and sharing of open data, will also help spread the knowledge that data can be shared before publication, without diluting academic credit and still allowing later publication. We encourage the community, particularly journal editors and reviewers, to support open science. Ultimately, we are all working in this field to find new medicines to help the millions of people infected with helminths, and data sharing and open science can only expedite this aim.
